# Amino Alcohol- (NPS-2143) and Quinazolinone-Derived Calcilytics (ATF936 and AXT914) Differentially Mitigate Excessive Signalling of Calcium-Sensing Receptor Mutants Causing Bartter Syndrome Type 5 and Autosomal Dominant Hypocalcemia

**DOI:** 10.1371/journal.pone.0115178

**Published:** 2014-12-15

**Authors:** Saskia Letz, Christine Haag, Egbert Schulze, Karin Frank-Raue, Friedhelm Raue, Benjamin Hofner, Bernhard Mayr, Christof Schöfl

**Affiliations:** 1 Division of Endocrinology and Diabetes, Department of Medicine I, Universitätsklinikum Erlangen, Erlangen, Germany; 2 Endocrine Practice, Heidelberg, Germany; 3 Department of Medical Informatics, Biometry and Epidemiology, Friedrich-Alexander-University Erlangen-Nuremberg, Erlangen, Germany; University of Bari Aldo Moro, Italy

## Abstract

**Introduction:**

Activating calcium sensing receptor (CaSR) mutations cause autosomal dominant hypocalcemia (ADH) characterized by low serum calcium, inappropriately low PTH and relative hypercalciuria. Four activating CaSR mutations cause additional renal wasting of sodium, chloride and other salts, a condition called Bartter syndrome (BS) type 5. Until today there is no specific medical treatment for BS type 5 and ADH. We investigated the effects of different allosteric CaSR antagonists (calcilytics) on activating CaSR mutants.

**Methods:**

All 4 known mutations causing BS type 5 and five ADH mutations were expressed in HEK 293T cells and receptor signalling was studied by measurement of intracellular free calcium in response to extracellular calcium ([Ca^2+^]_o_). To investigate the effect of calcilytics, cells were stimulated with 3 mM [Ca^2+^]_o_ in the presence or absence of NPS-2143, ATF936 or AXT914.

**Results:**

All BS type 5 and ADH mutants showed enhanced signalling activity to [Ca^2+^]_o_ with left shifted dose response curves. In contrast to the amino alcohol NPS-2143, which was only partially effective, the quinazolinone calcilytics ATF936 and AXT914 significantly mitigated excessive cytosolic calcium signalling of all BS type 5 and ADH mutants studied. When these mutants were co-expressed with wild-type CaSR to approximate heterozygosity in patients, ATF936 and AXT914 were also effective on all mutants.

**Conclusion:**

The calcilytics ATF936 and AXT914 are capable of attenuating enhanced cytosolic calcium signalling activity of CaSR mutations causing BS type 5 and ADH. Quinazolinone calcilytics might therefore offer a novel treatment option for patients with activating CaSR mutations.

## Introduction

The calcium-sensing receptor (CaSR) is a key regulator of calcium homeostasis and is expressed in parathyroid, kidney, bone and other organs involved in calcium metabolism [1]. Binding of calcium to the extracellular domain of the receptor results in conformational changes in the transmembrane domain, which in turn activates different G-proteins [2], [3]. The best-studied signalling pathway is activation of phospholipase C by G_q/11_ with a consequent rise in cytosolic free calcium ([Ca^2+^]_i_) [4], [5]. Activation of the receptor by rising serum calcium inhibits PTH secretion from parathyroid cells and increases calcium excretion by the kidney. This lowers serum calcium and completes the homeostatic calcium feedback loop [6].

Activating mutations of the CaSR disturb this regulatory feedback loop by lowering the calcium set-point of the CaSR and cause autosomal dominant hypocalcemia (ADH). ADH patients have mild to moderate hypocalcemia with an inappropriately normal or high urinary calcium excretion and low to normal PTH levels [7]. These patients suffer from tissue calcifications especially in the brain and kidney [8], [9] and sometimes show defects in bone mineralization [10]. Vitamin D and calcium supplementation is commonly used to raise serum calcium in ADH patients, but this does not correct the underlying molecular defect, often worsens hypercalciuria and promotes kidney stone formation or nephrocalcinosis. Treatment with PTH (1–34) increases serum calcium and reduces hypercalciuria but does not normalize urinary calcium excretion and does not prevent renal complications [10], [11].

Four activating mutations of the CaSR cause additional renal sodium, chloride and magnesium wasting which results in hyperreninemia, hyperaldosteronism, hypokalemia, and metabolic alkalosis, a condition called Bartter syndrome type 5 (BS type 5) [12]–[16]. The molecular basis for these different and distinct clinical phenotypes caused by activating CaSR mutations is unknown.

Since the synthesis of the first calcilytic compound NPS-2143 [17], [18] several allosteric antagonists of the CaSR from different chemical classes such as amino alcohols, diaminocyclohexanes, quinazolinones and benzimidazoles have been developed [19]. These calcilytics have the potential to directly correct the molecular cause of ADH and BS type 5. Structural and functional studies suggest common binding sites on the CaSR for amino alcohol and diaminocyclohexane calcilytics. Quinazolinone and benzimidazole calcilytics also share a common set of binding sites on the CaSR, which are however partly different from the bindings sites of amino alcohols and diaminocyclohexanes and set these compounds apart as a distinct group of calcilytics ([19] and references therein).

In this study we tested whether amino alcohol and quinazolinone calcilytics could mitigate excessive cytosolic calcium signal transduction of CaSR mutants leading to BS type 5 or ADH thus potentially providing a novel treatment option for these patients [19].

## Methods

### Expression of mutant CaSR in human embryonic kidney (HEK) 293T cells

Expression vectors for wild-type (wt) and mutant CaSR were generated by site-directed mutagenesis and transfected in HEK 293T cells cultured on glass coverslips [20]–[22]. One µg CaSR expression vector and 0.1 µg YFP expression vector mYF-C2 or for co-transfection experiments 0.5 µg mutant and 0.5 µg YFP-tagged wt-CaSR were used for transient transfection. All 4 known *CaSR* mutations that cause BS type 5 (K29E, L125P, C131W, A843E) have been reported before [12]–[16]. The ADH *CaSR* mutation A835D is novel and was found in a patient referred for endocrine evaluation of incidentally detected low serum calcium. The ADH mutants T151R, P221L, G830S and A844T have been described by us before [20].

### Measurement of [Ca^2+^]_i_ and effect of calcilytics

Transiently transfected HEK 293T cells were loaded with 5 µM Fura-2/AM (Invitrogen) and placed in superfusion buffer. Single cells of healthy appearance were selected by YFP fluorescence and used for calcium measurements by dual wavelength excitation microfluorometry. Dose response curves were carried out as described [20]. To determine the effect of calcilytics, cells were treated with 3 mM [Ca^2+^]_o_ for 5 min, 0.5 mM [Ca^2+^]_o_ for 3 min, control buffer with DMSO 0.1%, NPS-2143 (300 nM or 1 µM), ATF936 (300 nM) or AXT914 (300 nM) for 2 min, and a second stimulation with 3 mM [Ca^2+^]_o_ in the presence or absence of calcilytics for 5 min. Specificity of calcilytics and cell viability, was verified by a final stimulation with 10 mM [Ca^2+^]_o_ which can overcome inhibition by calcilytics [18]. NPS-2143 was obtained from Hangzhou Hetd Industry (Hangzhou, China), ATF936 and AXT914 were a gift from Novartis (Basel, Switzerland). All three substances were dissolved in dimethylsulfoxide (DMSO) and used at final concentration of 300 nM (NPS-2143, ATF936 and AXT914) and 1 µM (NPS-2143). The final DMSO concentration was kept constant at 0.1%.

### Statistics

Nonlinear regression of dose response curves was performed with GraphPad Prism 6 (GraphPad, San Diego, CA) using Δ[Ca^2+^]_i_ values. EC_50_ values and 95% confidence intervals were determined from the non-linear regression curves [20]. The regression fits of wt and mutant CaSR were tested for statistically significant differences with an F-test [23], [24] using GraphPad Prism 6 (GraphPad, San Diego, CA) by comparing two nested models. In the first model the parameters EC50 and maximum response were common for both wt and mutant CaSR and in the second model these parameters were allowed to be different. The effect of the calcilytics on the cytosolic calcium response caused by stimulation with 3 mM [Ca^2+^]_o_ was evaluated by Kruskal-Wallis one way ANOVA on ranks with Dunn's method for multiple comparisons versus control group with normalized Δ[Ca^2+^]_i_ signals using SigmaPlot version 11.0 (Systat, Erkrath, Germany). Values shown are mean ±95% confidence intervals.

## Results

### [Ca^2+^]_i_ signalling of CaSR mutants

**Figure 1:**
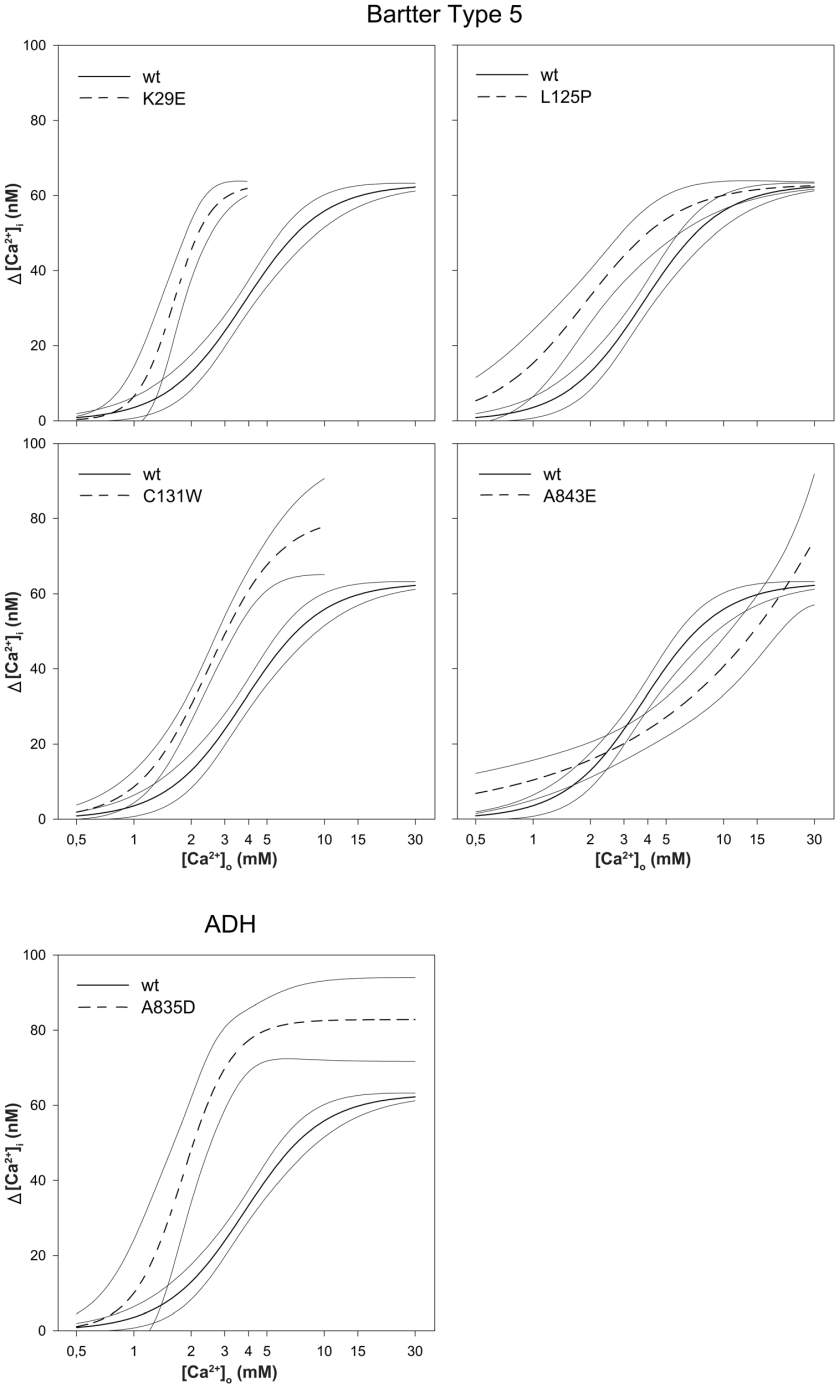
Sensitivity of BS type 5 mutants, the A835D ADH mutant and wt-CaSR, to [Ca^2+^]_o_. Dose-response curves with 95% confidence intervals of Δ[Ca^2+^]_i_ in response to a stepwise increase of [Ca^2+^]_o_ for BS type 5 and ADH mutants compared to wt-CaSR. Results from 11 to 27 individual cells measured in at least 4 independent experiments are shown.

All four BS type 5 *CaSR* mutations and the novel ADH mutant A835D showed activation of the cytosolic calcium pathway at lower [Ca^2+^]_o_ concentrations than wt-CaSR (Fig. 1) with significantly left shifted dose response curves (EC_50_ 1.4 mM to 2.3 mM, p<0.001–0.008 vs wt-CaSR, Table 1). The A843E CaSR mutant, however, which is the only BS type 5 mutation in the transmembrane domain known to date, did not display a classical sigmoidal dose response curve, which is consistent with previously published results [14], [16], [25]. Nevertheless, an exaggerated [Ca^2+^]_i_ response was present at [Ca^2+^]_o_ below 2.5 mM when compared to wt-CaSR.

**Table 1:** Results of the nonlinear regression analyses of dose-response-curves of intracellular free calcium in response to extracellular calcium.

Amino acid	Nucleotide			Regression Fit		EC_50_ [Ca^2+^]_o_ (mM)	
Change	Change	Location	Phenotype	p vs wt	R^2^	mean	95% CI
wt				-	0.76	3.29	2.99–3.62
K29E	A85G	ECD	BS type 5	<0.001	0.96	1.42	1.33–1.51
L125P	T374C	ECD	BS type 5	<0.001	0.67	1.70	1.47–1.97
C131W	C393G	ECD	BS type 5	<0.001	0.91	1.57	1.43–1.72
A843E	C2528A	TM7	BS type 5	0.008	0.64	2.34	1.88–2.93
A835D	C2504A	ECL3	ADH	<0.001	0.69	2.02	1.79–2.28

wt, wild type CaSR; EC_50_ [Ca^2+^]_o_, extracellular calcium concentration giving half maximal response determined from normalized data; 95% CI, 95% confidence interval. The p-values for the regression fit was obtained by comparing nested models with EC_50_ and maximum response common between mutant and wildtype or allowed to be different. TM7, transmembrane domain 7; ECD, extracellular domain; ECL3, extracellular loop 3.

### Effect of amino alcohol (NPS-2143) and quinazolinone (ATF936 and AXT914) calcilytics

**Figure 2:**
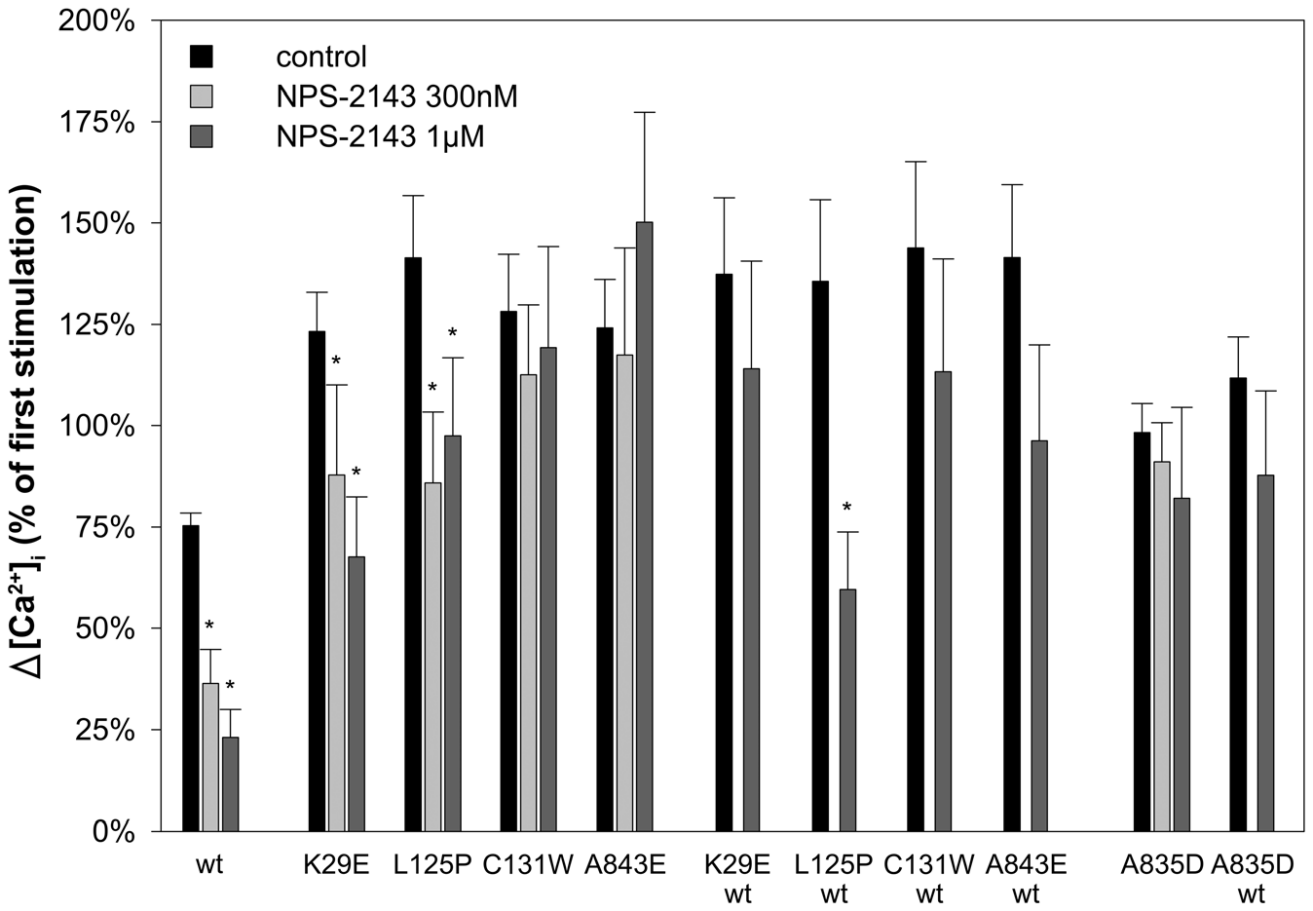
Effect of the amino alcohol calcilytic NPS-2143 on CaSR mutants causing BS type 5 or ADH (A835D). Δ[Ca^2+^]_i_ in response to stimulation with 3 mM [Ca^2+^]_o_ with and without NPS-2143 treatment normalized to the first stimulation with 3 mM [Ca^2+^]_o_ (±95% confidence interval) in HEK 293T cells transfected with wt-CaSR, BS type 5 or ADH CaSR mutants and co-transfected with wt and mutant CaSR as indicated. Results from 9 to 205 individual cells measured in at least 4 independent experiments are shown. *, P<0.05 for the effect of calcilytics vs. control (DMSO) on the [Ca^2+^]_o_-induced increase in [Ca^2+^]_i_ as determined by Kruskal-Wallis one way ANOVA on ranks with Dunn's method for multiple comparisons.

**Figure 3:**
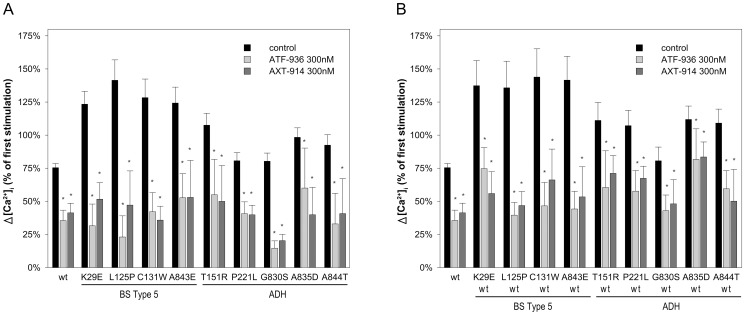
Effect of quinazolinone calcilytics on BS type 5 and ADH CaSR mutants. Δ[Ca^2+^]_i_ in response to stimulation with 3 mM [Ca^2+^]_o_ with and without ATF936 and AXT914 treatment normalized to the first stimulation with 3 mM [Ca^2+^]_o_ (±95% confidence interval) in HEK 293T cells transfected with wt or mutant CaSR (A) and co-transfected with wt and mutant CaSR (B). Results from 7 to 205 individual cells measured in at least 3 independent experiments are shown. *, P<0.05 for the effect of calcilytics vs. control (DMSO) on the [Ca^2+^]_o_-induced increase in [Ca^2+^]_i_ as determined by Kruskal-Wallis one way ANOVA on ranks with Dunn's method for multiple comparisons.

To test whether the amino alcohol calcilytic NPS-2143 and the quinazolinones ATF936 and AXT914 could mitigate the exaggerated cytosolic calcium response of BS type 5 and ADH CaSR mutants cells were stimulated twice with 3 mM [Ca^2+^]_o_. Cells were first stimulated with 3 mM [Ca^2+^]_o_ for 5 min, then treated for 5 min with 0.5 mM [Ca^2+^]_o_ followed by a second 5 min stimulation with 3 mM [Ca^2+^]_o_. Two min before and throughout the second stimulation cells were perfused with medium containing NPS-2143, ATF936, AXT914, or 0.1% DMSO (control). The first stimulation was used for internal normalization to evaluate the effect of the calcilytics. In cells transfected with wt-CaSR and ADH mutants the [Ca^2+^]_i_ response to the second [Ca^2+^]_o_ stimulus was similar or lower when compared to the first [Ca^2+^]_i_ response. Remarkably, however, cells transfected with BS type 5 CaSR mutants displayed a significantly higher second [Ca^2+^]_i_ response (p<0.05 - p<0.001) (Figs. 2 and 3).

NPS-2143 (300 nM and 1 µM) significantly attenuated the [Ca^2+^]_o_-induced [Ca^2+^]_i_ response of two BS type 5 mutants (K29E, L125P). Interestingly, the CaSR mutant K29E lost responsiveness to NPS-2143 when co-expressed with wt-receptor to approximate heterozygosity in patients (Fig. 2). The novel ADH CaSR mutant A835D either expressed alone or co-expressed with wt-CaSR was unresponsive to NPS-2143 up to 1 µM (Fig. 2). These data are consistent with our previous observation that NPS-2143 can reduce excessive signal transduction of some but not all ADH CaSR mutants [20].

In contrast, as depicted in Fig. 3 both ATF936 and AXT914 at 300 nM significantly mitigated excessive signalling of all BS type 5 CaSR and all ADH mutants including four ADH mutants that had been previously tested by us with NPS-2143 [20]. The quinazolinones also inhibited [Ca^2+^]_o_–induced [Ca^2+^]_i_ signalling in cells co-expressing both mutant and wt-CaSR (Fig. 3B). The [Ca^2+^]_i_ response to 3 mM [Ca^2+^]_o_ was lowered to a degree comparable to control treated cells expressing wt-CaSR only.

## Discussion

Gain-of-function mutations of the CaSR either cause autosomal dominant hypercalcemia (ADH), which is characterized by hypocalcemia and hypercalciuria, or BS type 5, which in addition is associated with renal salt wasting leading to hyperreninemia, hyperaldosteronism and hypokalemia [26], [27]. The molecular background for these different phenotypes is unknown.

In the present study we investigated all four CaSR mutations that cause BS type 5 known to date (K29E, L125P, C131W in the extracellular domain and A843E in the transmembrane region 7) and five CaSR mutations causing ADH located in the same regions. Consistent with the clinical phenotypes all *CaSR* mutations inadequately activate the cytosolic calcium signalling pathway as demonstrated by activation of this pathway even at extracellular calcium concentrations below 2 mM (Fig. 1) and [20]. CaSR K29E showed the lowest EC_50_ of all BS type 5 mutants, but causes only a rather mild phenotype in the affected patients [12], [15] suggesting that the severity of the clinical symptoms in BS type 5 may not be correlated with the degree of calcium signalling pathway activation. A further difference between BS type 5 and ADH causing CaSR mutants was observed in experiments with repeated [Ca^2+^]_o_ stimulation. BS type 5 CaSR mutants but not ADH mutants demonstrated an enhanced second [Ca^2+^]_i_ response (Figs. 2 and 3). This suggests that the calcium and possibly other signalling mechanisms may be altered differentially between BS type 5 and ADH. Elucidating these mechanisms may help to identify new therapeutic targets for BS type 5 and ADH patients.

Novel treatment strategies are indeed needed for both patient groups. Current treatment is primarily symptomatic. To treat hypocalcemia vitamin D, calcium and thiazide diuretics are used. Symptom relief is often inadequate and therapy is limited by increased urinary calcium excretion which further raises the risk of kidney stone formation or nephrocalcinosis [8], [9], [14], [28]. PTH replacement with teriparatide is another option to raise serum calcium, but inadequately high calcium excretion persists [10], [11]. In patients with BS type 5 electrolyte replacement, amiloride or spironolactone may be used to control hypokalemia. None of these therapeutic strategies, however, target the underlying molecular defect and adequately correct altered calcium and electrolyte metabolism.

Inhibitors of CaSR function called calcilytics may provide a novel therapeutic approach. They have been developed to promote PTH secretion in osteoporotic patients with the aim to increase bone formation similar to teriparatide [18], [19], [29]. Although calcilytics are not yet approved for therapeutic use, phase II trials of AXT-914 and the NPS-2143 derivative ronacaleret in healthy subjects and osteopenic postmenopausal women demonstrated an increase in serum calcium [29]–[31]. In studies where it was examined a reduction in urinary calcium excretion was observed [30], which would be the desired effect in ADH and BS type 5 patients. These drugs were well tolerated in the phase II trials. The most common adverse effects were mild disorders of the gastrointestinal tract and nervous system such as fatigue, headache, constipation, diarrhea, nausea and dyspepsia [29], [31], which might be related to CaSR expressed in gut [32] and brain [33]. As CaSR is also present in skin, lung, heart, mammary glands and numerous other tissues (reviewed in [27]) further short- and long-term adverse effects of calcilytics cannot be ruled out. In ADH and BS5 patients, however, calcilytics would not decrease normal CaSR function, but would restore normal function of an exaggerated CaSR activity. Nevertheless, short- and long-term adverse events may occur and should therefore be carefully monitored, when these compounds are used in clinical practice.

In this study we tested NPS-2143 for the first time on *CaSR* mutations that cause BS type 5. Only two of 4 known CaSR mutants were sensitive to NPS-2143 either expressed alone or when co-expressed with wt-CaSR to approximate heterozygosity in patients. This resembles previous data from us and others showing that some but not all ADH CaSR mutants were sensitive to NPS-2143 [20], [34], [35]. Thus, amino alcohol derived calcilytics may not be the ideal drugs to treat disorders caused by activating CaSR mutations. There are CaSR antagonists with different chemical structures such as diaminocyclohexanes, quinazolinones and benzimidazoles. Studies with mutated CaSR and molecular modelling suggest that the binding sites for amino alcohols and diaminocyclohexanes largely overlap, but that these binding sites are at least partly distinct from those for quinazolinones and benzimidazoles ([19] and references therein). This might explain the differential effects of NPS-2143 and ATF936 or AXT914 on excessive signalling of the ADH CaSR mutant A835D, as amino alcohols, but not quinazolinones form a hydrogen bond to amino acid E837 of the CaSR ([19] and references therein).

The quinazolinones ATF936 and AXT914 in contrast to NPS-2143 significantly mitigated excessive cytosolic calcium signalling of all tested BS type 5 and ADH CaSR mutants when expressed alone or co-expressed with wt-CaSR. Both compounds could suppress the calcium signalling activity of the activating CaSR mutants to levels of wt-CaSR and they were efficacious on receptor proteins with mutations in the extracellular and transmembrane region. Recently, the novel ADH CaSR mutant D410E has also been tested and found to be sensitive to AXT914 in vitro [36]. Taken together, the quinazolinone calcilytics tested here could be a promising new therapeutic approach for ADH and BS type 5 patients.

Here we studied the calcium signalling pathway, which appears to be involved in the regulation of key processes of calcium metabolism [1], [5]. However, CaSR couples via different G-proteins to a number of other intracellular signalling events [2], [3] and it is yet unknown by which signalling mechanisms CaSR regulates renal salt handling. It is therefore difficult to predict the impact of quinazolinones calcilytics on hypercalciuria and renal salt wasting in patients with activating mutations, although the amino alcohol ronacaleret reduced urinary calcium excretion in healthy probands with wt-CaSR [30]. The quinazolinone AXT914 was also well tolerated and increased serum calcium in phase II trials, but its effects on the urinary excretion of calcium and other ions have not been reported [29]. The answer will have to await the availability of calcilytic drugs for clinical treatment of patients with BS type 5 and ADH.

## References

[1] Riccardi D, Brown EM (2010) Physiology and pathophysiology of the calcium-sensing receptor in the kidney. Am J Physiol Renal Physiol 298:F485–499. 19923405 10.1152/ajprenal.00608.2009 PMC2838589

[2] Hu J, Spiegel AM (2007) Structure and function of the human calcium-sensing receptor: insights from natural and engineered mutations and allosteric modulators. J Cell Mol Med 11:908–922. 17979873 10.1111/j.1582-4934.2007.00096.x PMC4401263

[3] Brown EM, Gamba G, Riccardi D, Lombardi M, Butters R, et al (1993) Cloning and characterization of an extracellular Ca(2+)-sensing receptor from bovine parathyroid. Nature 366:575–580. 8255296 10.1038/366575a0

[4] Hofer AM, Brown EM (2003) Extracellular calcium sensing and signalling. Nat Rev Mol Cell Biol 4:530–538. 12838336 10.1038/nrm1154

[5] Nesbit MA, Hannan FM, Howles SA, Babinsky VN, Head RA, et al (2013) Mutations affecting G-protein subunit alpha11 in hypercalcemia and hypocalcemia. N Engl J Med 368:2476–2486. 23802516 10.1056/NEJMoa1300253 PMC3773604

[6] Egbuna OI, Brown EM (2008) Hypercalcaemic and hypocalcaemic conditions due to calcium-sensing receptor mutations. Best Pract Res Clin Rheumatol 22:129–148. 18328986 10.1016/j.berh.2007.11.006 PMC2364635

[7] Pollak MR, Brown EM, Estep HL, McLaine PN, Kifor O, et al (1994) Autosomal dominant hypocalcaemia caused by a Ca(2+)-sensing receptor gene mutation. Nat Genet 8:303–307. 7874174 10.1038/ng1194-303

[8] Raue F, Pichl J, Dörr HG, Schnabel D, Heidemann P, et al (2011) Activating mutations in the calcium-sensing receptor: genetic and clinical spectrum in 25 patients with autosomal dominant hypocalcaemia - a German survey. Clin Endocrinol (Oxf) 75:760–765. 21645025 10.1111/j.1365-2265.2011.04142.x

[9] Sayer JA, Pearce SH (2003) Extracellular calcium-sensing receptor dysfunction is associated with two new phenotypes. Clin Endocrinol (Oxf) 59:419–421. 14510901 10.1046/j.1365-2265.2003.01869.x

[10] Theman TA, Collins MT, Dempster DW, Zhou H, Reynolds JC, et al (2009) PTH(1–34) replacement therapy in a child with hypoparathyroidism caused by a sporadic calcium receptor mutation. J Bone Miner Res 24:964–973. 19063686 10.1359/JBMR.081233 PMC2672210

[11] Gonzales MC, Lieb DC, Richardson DW, O'Brian JT, Aloi JA, et al (2013) Recombinant human parathyroid hormone therapy (1–34) in an adult patient with a gain-of-function mutation in the calcium-sensing receptor-a case report. Endocr Pract 19:e24–28. 23186954 10.4158/EP12132.CR

[12] Hu J, Mora S, Weber G, Zamproni I, Proverbio MC, et al (2004) Autosomal dominant hypocalcemia in monozygotic twins caused by a de novo germline mutation near the amino-terminus of the human calcium receptor. J Bone Miner Res 19:578–586. 15005845 10.1359/JBMR.040106

[13] Vargas-Poussou R, Huang C, Hulin P, Houillier P, Jeunemaitre X, et al (2002) Functional characterization of a calcium-sensing receptor mutation in severe autosomal dominant hypocalcemia with a Bartter-like syndrome. J Am Soc Nephrol 13:2259–2266. 12191970 10.1097/01.asn.0000025781.16723.68

[14] Watanabe S, Fukumoto S, Chang H, Takeuchi Y, Hasegawa Y, et al (2002) Association between activating mutations of calcium-sensing receptor and Bartter's syndrome. Lancet 360:692–694. 12241879 10.1016/S0140-6736(02)09842-2

[15] Vezzoli G, Arcidiacono T, Paloschi V, Terranegra A, Biasion R, et al (2006) Autosomal dominant hypocalcemia with mild type 5 Bartter syndrome. J Nephrol 19:525–528. 17048213

[16] Zhao XM, Hauache O, Goldsmith PK, Collins R, Spiegel AM (1999) A missense mutation in the seventh transmembrane domain constitutively activates the human Ca2+ receptor. FEBS Lett 448:180–184. 10217436 10.1016/s0014-5793(99)00368-3

[17] Gowen M, Stroup GB, Dodds RA, James IE, Votta BJ, et al (2000) Antagonizing the parathyroid calcium receptor stimulates parathyroid hormone secretion and bone formation in osteopenic rats. J Clin Invest 105:1595–1604. 10841518 10.1172/JCI9038 PMC300853

[18] Nemeth EF, Delmar EG, Heaton WL, Miller MA, Lambert LD, et al (2001) Calcilytic compounds: potent and selective Ca2+ receptor antagonists that stimulate secretion of parathyroid hormone. J Pharmacol Exp Ther 299:323–331. 11561095

[19] Widler L (2011) Calcilytics: antagonists of the calcium-sensing receptor for the treatment of osteoporosis. Future Med Chem 3:535–547. 21526895 10.4155/fmc.11.17

[20] Letz S, Rus R, Haag C, Dörr HG, Schnabel D, et al (2010) Novel activating mutations of the calcium-sensing receptor: the calcilytic NPS-2143 mitigates excessive signal transduction of mutant receptors. J Clin Endocrinol Metab 95:E229–233. 20668040 10.1210/jc.2010-0651

[21] Rus R, Haag C, Bumke-Vogt C, Bähr V, Mayr B, et al (2008) Novel inactivating mutations of the calcium-sensing receptor: the calcimimetic NPS R-568 improves signal transduction of mutant receptors. J Clin Endocrinol Metab 93:4797–4803. 18796518 10.1210/jc.2008-1076

[22] Szczawinska D, Schnabel D, Letz S, Schöfl C (2014) A homozygous CaSR mutation causing a FHH phenotype completely masked by vitamin D deficiency presenting as rickets. J Clin Endocrinol Metab epub ahead of print. 10.1210/jc.2013-3593 24517148

[23] Motulsky H, Christopoulos A (2004) Fitting Models to Biological Data Using Linear and Nonlinear Regression: A Practical Guide to Curve Fitting: Oxford University Press, USA.

[24] Bates DM, Watts DG (1998) Nonlinear Regression Analysis and Its Applications. New York: Wiley.

[25] Leach K, Wen A, Davey AE, Sexton PM, Conigrave AD, et al (2012) Identification of molecular phenotypes and biased signaling induced by naturally occurring mutations of the human calcium-sensing receptor. Endocrinology 153:4304–4316. 22798347 10.1210/en.2012-1449

[26] D'Souza-Li L (2006) The calcium-sensing receptor and related diseases. Arq Bras Endocrinol Metabol 50:628–639. 17117288 10.1590/s0004-27302006000400008

[27] Magno AL, Ward BK, Ratajczak T (2011) The calcium-sensing receptor: a molecular perspective. Endocr Rev 32:3–30. 20729338 10.1210/er.2009-0043

[28] Lienhardt A, Bai M, Lagarde JP, Rigaud M, Zhang Z, et al (2001) Activating mutations of the calcium-sensing receptor: management of hypocalcemia. J Clin Endocrinol Metab 86:5313–5323. 11701698 10.1210/jcem.86.11.8016

[29] John MR, Harfst E, Loeffler J, Belleli R, Mason J, et al (2014) AXT914 a novel, orally-active parathyroid hormone-releasing drug in two early studies of healthy volunteers and postmenopausal women. Bone 64C:204–210. 10.1016/j.bone.2014.04.015 24769332

[30] Caltabiano S, Desjardins J, Hossain M, Kurtinecz M, Fitzpatrick L (2009) Characterization of the effect of ronacaleret, a calcium-sensing receptor antagonist, on renal calcium excretion. J Bone Miner Res 24 Suppl 1. 10.1016/j.bone.2013.05.021 23756230

[31] Fitzpatrick L, Dabrowski C, Cicconetti G, Papapoulos S, Bone H, et al (2009) Ronacaleret, a calcium-sensing receptor antagonist: results of a 1 year double-blind, placebo-controlled, dose-ranging phase II study. J Bone Miner Res 24 Suppl 1.

[32] Macleod RJ (2013) CaSR function in the intestine: Hormone secretion, electrolyte absorption and secretion, paracrine non-canonical Wnt signaling and colonic crypt cell proliferation. Best Pract Res Clin Endocrinol Metab 27:385–402. 23856267 10.1016/j.beem.2013.05.005

[33] Liu XL, Lu YS, Gao JY, Marshall C, Xiao M, et al (2013) Calcium sensing receptor absence delays postnatal brain development via direct and indirect mechanisms. Mol Neurobiol 48:590–600. 23564401 10.1007/s12035-013-8448-0

[34] Lia-Baldini AS, Magdelaine C, Nizou A, Airault C, Salles JP, et al (2013) Two novel mutations of the calcium-sensing receptor gene affecting the same amino acid position lead to opposite phenotypes and reveal the importance of p.N802 on receptor activity. Eur J Endocrinol 168:K27–34. 23169696 10.1530/EJE-12-0714

[35] Hu J, McLarnon SJ, Mora S, Jiang J, Thomas C, et al (2005) A region in the seven-transmembrane domain of the human Ca2+ receptor critical for response to Ca2+. J Biol Chem 280:5113–5120. 15591042 10.1074/jbc.M413403200

[36] Park SY, Mun HC, Eom YS, Baek HL, Jung TS, et al (2013) Identification and characterization of D410E, a novel mutation in the loop 3 domain of CASR, in autosomal dominant hypocalcemia and a therapeutic approach using a novel calcilytic, AXT914. Clin Endocrinol (Oxf) 78:687–693. 23009664 10.1111/cen.12056

